# Digitizing death reporting in Zambia: evaluation of a mobile-based civil registration system

**DOI:** 10.1080/16549716.2026.2732479

**Published:** 2026-09-28

**Authors:** Collins Ochieng Odhiambo, Esther Nyadzua Katama, Brian Munkombwe, Chomba Mwango, Saviour Moyo, Christopher Chabila Mapoma

**Affiliations:** a Bloomberg Data for Health Initiative-CDC Foundation, Civil Registration and Vital Statistics department, Kisumu, Kenya; b Department of Program and Innovation office, CDC Foundation, Atlanta, GA, USA; c Bloomberg Data for Health Initiative-CDC Foundation, Civil Registration and Vital Statistics department, Lusaka, Zambia; d Ministry of Health, ICT Mortality Surveillance department, Lusaka, Zambia; e Department of Population Studies, School of Humanities and Social Sciences, University of Zambia, Lusaka, Zambia

**Keywords:** Digital health, vital statistics, civil registration, mortality, KoboToolbox

## Abstract

**Background:**

Strengthening Civil Registration and Vital Statistics (CRVS) systems is essential for generating reliable evidence to guide population health policy and planning. Routine death reporting is a critical first step toward improving data for effective health interventions in low- and middle-income countries.

**Objectives:**

This study evaluates the operational effectiveness of a mobile electronic reporting (e-reporting) system in Zambia, assessing improvements in timeliness, completeness and data quality compared to the paper-based process.

**Methods:**

An implementation case study was conducted in burial offices across high-mortality provinces (Lusaka, Central, Copperbelt, and Southern Provinces) and districts in Zambia. An electronic version of Form 13, the official death notification form, was developed using KoboToolbox to capture facility and community deaths in real time. A dashboard provided real-time monitoring and analysis.

**Results:**

Over six months, the electronic platform captured 115,573 deaths that occurred between January 2021 and June 2025. The e-reporting system achieved an estimated completeness rate of 17.4% (95% CI:17.3%−17.5%), statistically significantly higher than the manual system’s completeness rate of 11.9% (95% CI: 11.8%−12.0%) (chi2 = 4.01, *p* < 0.045). Reporting delays also reduced from an average of 6 days (manual system) to 3 days (e-reporting; *p* < 0.001). Completing a form electronically took an average of 4.6 minutes (median: 4.0).

**Conclusions:**

Digitizing CRVS death reporting in Zambia improved timeliness, completeness, and data quality, enabling rapid analysis and policy response. These findings demonstrate the potential of digital platforms to strengthen CRVS, enhance disease surveillance, and support data-driven health interventions.

## Paper Context

- **Main findings**: Integrating electronic data collection and analysis platforms into Civil Registration and Vital Statistics systems significantly improved death notification completeness, timeliness and reduced reporting delays, demonstrating measurable gains in system performance.
- **Added knowledge**: This work provides operational evidence that low-cost, open-source digital platforms integrated with real-time data capture and quality checks can strengthen Civil Registration and Vital Statistics functions in resource-limited settings.
- **Global health impact for policy and action**: Scaling such cost-effective digital reporting systems can enhance timely mortality data availability, supporting evidence-based decision-making and more responsive public health interventions in low- and middle-income countries.

## Background

Civil Registration and Vital Statistics (CRVS) death reporting and notification are essential for accurately documenting mortality in any country. Reliable CRVS records and the resulting statistics underpin national health and population policies, enabling governments to respond to emerging epidemics [1]. Despite this importance, the World Health Statistics reveal that many low- and middle-income countries (LMICs), including Zambia, have low death registration rates and/or produce incomplete or unreliable vital statistics [2]. Zambia’s national death registration completeness rate is estimated to be below 15% [3,4], significantly limiting the ability to track disease burden and evaluate program impact. Zambia has increased death registration coverage from 20.6% in 2017 [5] to approximately 40.4% in 2022 [6], timeliness remains a challenge, reducing the usefulness of mortality data for surveillance and decision-making. Although improving, death registration completeness levels remain below the 50% target set in the Second National Strategic Action Plan for CRVS (2021–2025). As a result, delays continue to make CRVS data unusable for mortality surveillance; limit Zambia’s capacity to identify causes of death and timely health system response, while further hindering the achievement of SDGs. Strengthening death registration is therefore a critical public health priority explicitly targeted by the United Nations Sustainable Development Goal 16 [7].

In well-developed CRVS systems, digitization supports the collection of high-quality, timely mortality data and facilitates prompt notification of deaths [8,9]. However, many developing countries such as Zambia face a higher burden of disease and mortality without robust systems in place to ensure timely, accurate death reporting [3,4,10,11]. In Zambia, reliance on traditional paper-based methods leads to persistent operational challenges such as reporting delays, incomplete records, and lost forms, which undermines mortality surveillance [4,12]. Timely death reporting is particularly important for detecting sudden mortality trends and informing health outcome policies. According to Zambia’s Births and Deaths Registration Act [13], the legal framework stipulates that a death should be notified within 14 days and registered within 30 days to be considered timely. Registrations completed beyond 30 days but within 12 months are classified as late registrations, while those occurring after 12 months are considered delayed registrations. For the purposes of this study, timeliness and completeness were assessed against these statutory definitions.

In Zambia, the Department of National Registration, Passport and Citizenship (DNRPC) under the Ministry of Home Affairs and Internal Security (MoHAIS), manages all CRVS activities, currently relying on a manual paper-based system [*See Supplementary Figure 1 for schematic flow of manual death notification and registration in Zambia*]. Although some deaths are notified in a timely manner, delays occur in the subsequent transmission of notification documents from the health system to the Civil Registration Authority (CRA) for official registration into the central Integrated National Registration and Identification System (INRIS). This process is slow, costly, and inefficient, delaying the production of vital statistics reports. In cases where notification forms are submitted directly to the CRA, additional administrative constraints persist. These include limited staffing levels in some registration offices and competing operational demands, such as the redeployment of personnel to conduct national identification (ID) activities. As a result, death registration functions may be temporarily deprioritized, leaving cases unprocessed.

While the World Health Organization (WHO) recommends using mobile technologies to standardize mortality reporting [14] there is limited published operational evidence detailing the implementation efficiency, effectiveness, and logistical feasibility of integrating such digital tools into routine, high-volume CRVS operations within a government-mandated system in Zambia.

To address this gap, a mobile digital platform was implemented to capture high-quality mortality data in real time. This digital implementation case study compares the timeliness, completeness, and accuracy of demographic information in death notifications processed through the new electronic system with those reported via the existing manual method. It further highlights how digitization can streamline workflows and improve coordination between the civil registration and health sectors.

## Methods

### Study design and setting

This was an implementation case study comparing operational performance indicators before and after the introduction of electronic notification system. The study examined the routine death notification process in provinces with historically high volumes of death reports (Lusaka, Central, Copperbelt, and Southern Provinces) and substantial backlogs of unprocessed forms (deaths between 2021 and 2023). The implementation involved 16 sites, comprising district burial offices and corresponding health facilities, with an average of 2–3 officers per site from DNRPC and the MoH.

The e-notification was used both to retrospectively enter previously completed paper Form 13s from deaths occurring between 2021 and 2023 and to capture new deaths through the electronic notification process. Historical data for 2021–2023 were used as a reference for the traditional reporting process. The historical and electronic reporting periods were not strictly contemporaneous thus comparisons were interpreted as descriptive operational comparisons rather than as a causal pre-post evaluation.

### Interventions

With technical support from the Bloomberg Philanthropies Data for Health (D4H) initiative and in collaboration with the DNRPC, the project team implemented the following key actions:

1. Designing an electronic, mobile-based version of the official death notification form (Form 13)
2. Training registration and health officers in the use of the tool to support data entry, form archiving, and improved coordination with local council offices to facilitate the transfer of notification forms from health facilities.

### Electronic death reporting (e-reporting)

Form 13 – the official death notification document – was digitized using KoboToolbox [15] and hosted on the Ministry of Health (MoH) servers.

### Data validation and staff training

The digital tool integrated electronic validation checks to flag inconsistent or erroneous entries, such as non-plausible age entries (e.g. age > 120 years) or date inconsistencies, ensuring immediate data quality assurance. Training sessions for DNRPC officers and district-level staff included both didactic instruction and practical hands-on exercises, followed by a post-training competency assessment to ensure accurate system usage and high data quality standards.

The MoH assigned mortality surveillance officers to health facilities located near mortuaries. These officers were tasked with recording all deaths, including those occurring in the facility and those brought in from the community. For community deaths, before the body is admitted to the mortuary, officers assist burial officials and the deceased’s family in completing the paper Form 13. The officer then entered the details into the digital platform, scanned supporting documents, and uploaded the cause-of-death information. Officers were required to sync their entries with the main server by the end of each day to ensure timely submission.

After completing Form 13, the family would proceed to the burial office with the form, the deceased’s documents, and any required legal paperwork. The burial officer records the death in the official register, files the documents, and issues a burial permit upon payment of the fee. This permit number is later used to obtain the official death certificate.

To address the backlog of Form 13 submissions from 2021 to 2023, district and provincial offices sent all completed forms to the DNRPC. The D4H technical team supervised and managed the allocation of these forms for data entry. At the same time, notification officers captured new deaths from 2024 while conducting verbal autopsies on community deaths [11].

### Dashboard development

A central feature of the e-reporting system was the creation of a real-time death reporting dashboard. This tool served two main purposes:

1. Monitoring progress – visualizing the flow of death reports across provinces in real time.
2. Ensuring data quality – integrating built-in validation checks to automatically flag inconsistent data at the point of entry.

The dashboard, shown in Figure 1, ensured decision-makers had timely, high-quality data for public health planning and response.

**Figure 1.**
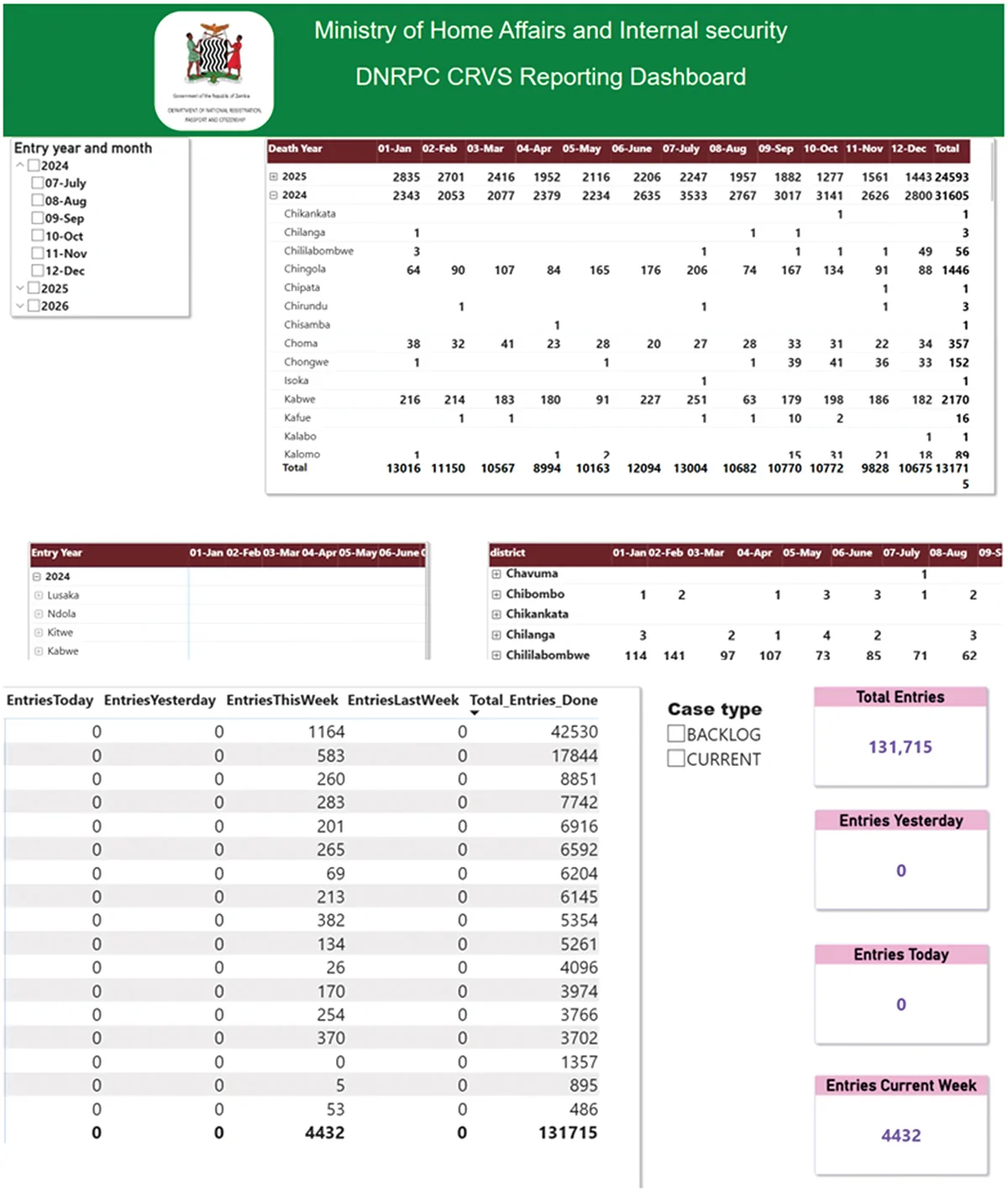
The different dashboard pages, highlighting key indicators being tracked. These include, among others, the number of deaths by year, province, and district; weekly and daily data collection progress; and the distribution of deaths by age and sex, with filters available by year, province, and district. The dashboard shown is not an official government website but a demonstration product developed during the initiative with Data for Health support. Permission to use this image was obtained from the relevant authorities as part of the approved scope of the project.

### Statistical analysis

Data from the digital platform was exported to R for statistical analysis. Death data from the manual registration system for the years 2021–2023 was provided by the DNRPC for comparison with the electronic reporting system.

Completeness was calculated using the standardized demographic approach: the proportion of total deaths notified during the study period (numerator) divided by the estimated total deaths (denominator). The denominator was derived from 2022 census data using the official Crude Death Rate (CDR) to estimate expected mortality. This approach assumes that the census derived CDR provides a reliable estimate of mortality at the population level. Limitations may include potential under- or over-estimation if the CDR does not fully capture sub-national variations, demographic shifts, or pandemic related mortality changes. Nonetheless, this method is widely used in CRVS assessments to provide a standardized denominator for completeness estimation.

$$\mathrm{Completeness}=\frac{\mathrm{Total}\;\;\mathrm{deaths}\;\;\mathrm{notified}}{\mathrm{Estimated}\;\;\mathrm{total}\;\;\mathrm{deaths}}\;\;\times \;\;100$$

Where the expected number of deaths was calculated as:

Expecteddeaths=Population×CrudeDeathRate

Timeliness was measured as the number of days from the date of death to the date of electronic entry. Backlogged e-reporting entries were excluded to prevent bias, and only deaths reported within 30 days were analyzed.

Timeliness (in days) was calculated as: Dateofentry−Dateofdeath

As this is a routine implementation case study examining system performance, formal sample size justification is not applicable; we analyzed all deaths notified within the operational period. Age and sex distributions were compared between e-reporting and traditional manual reporting records using Chi-square tests. Completeness and timeliness estimates were presented with 95% Confidence Intervals (CIs) and associated *p*-values. All analyses were performed in R v4.2.1.

## Results

Between 1 July 2024, and 10 June 2025, a total of 115,573 deaths that occurred between January 2021 and June 2025 were captured via the electronic notification system. Of these, 68,389 (59.2%) were male, with a median age at death of 40 years (IQR 17–62) (Table 1). Deaths among children under one year were the most common (18,884; 16.3%), while the 5–9 years (1,977; 1.7%) and 10–14 years (1,758; 1.5%) age groups had the fewest cases (Supplementary Table 1). The age-sex distribution mirrored patterns typical of developing countries – high early childhood mortality, lower mortality during adolescence, and rising mortality between ages 20–49 (particularly among males), followed by higher female mortality in older age groups (Figure 2). Spatially, Lusaka Province accounted for the majority of reported deaths (59.8%) (Supplementary Table 1). On average, completing a digital form took 4.6 minutes (median 4.0, IQR 2.9–5.7 minutes).

**Figure 2.**
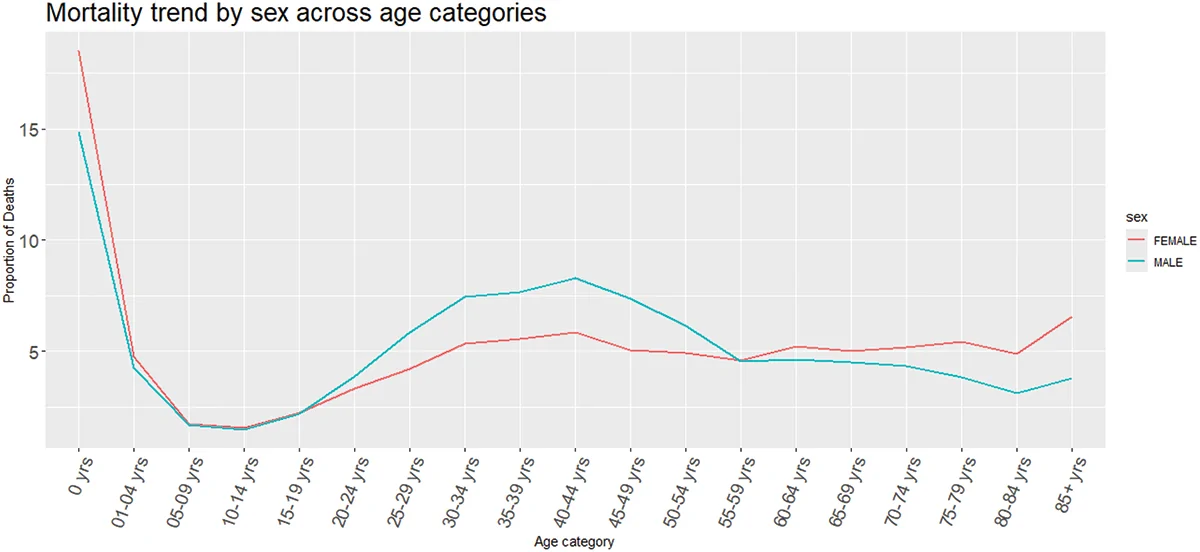
Percentage distribution of deaths captured through the electronic notification system by age categories and sex for the period of 2021–01 up to 2025–06.

**Table 1.** Demographic characteristics of deaths captured through the e-reporting system in Zambia, July 2024−June 2025.

Characteristics	*N* = 115573	
	n	%
Sex		
Male	68,389	59.2
Female	47,184	40.8
Total	115573	100
Age (years)		
Median (IQR)	40 (17–62)	–

### Comparison between deaths captured: e-reporting vs. manual system

#### Distribution of deaths

A significantly higher number of deaths were recorded by the electronic reporting system compared with those captured by the manual system submitted through INRIS (χ2 (1) = 4.01, *p *= <0.045) (Figure 3). This illustrates that the manual national registration system misses many deaths. If integrated with the national system, the e-reporting platform can complement it by capturing deaths that would otherwise go unregistered, therefore supplying additional, timely death reports hence improving completeness and reducing delays. The delayed entry of backlogged cases in the manual process has caused lack of availability of data for 2024 and 2025. This calls for efforts for continuous improvement and enhancement of such positive collaborations for process improvements.

**Figure 3.**
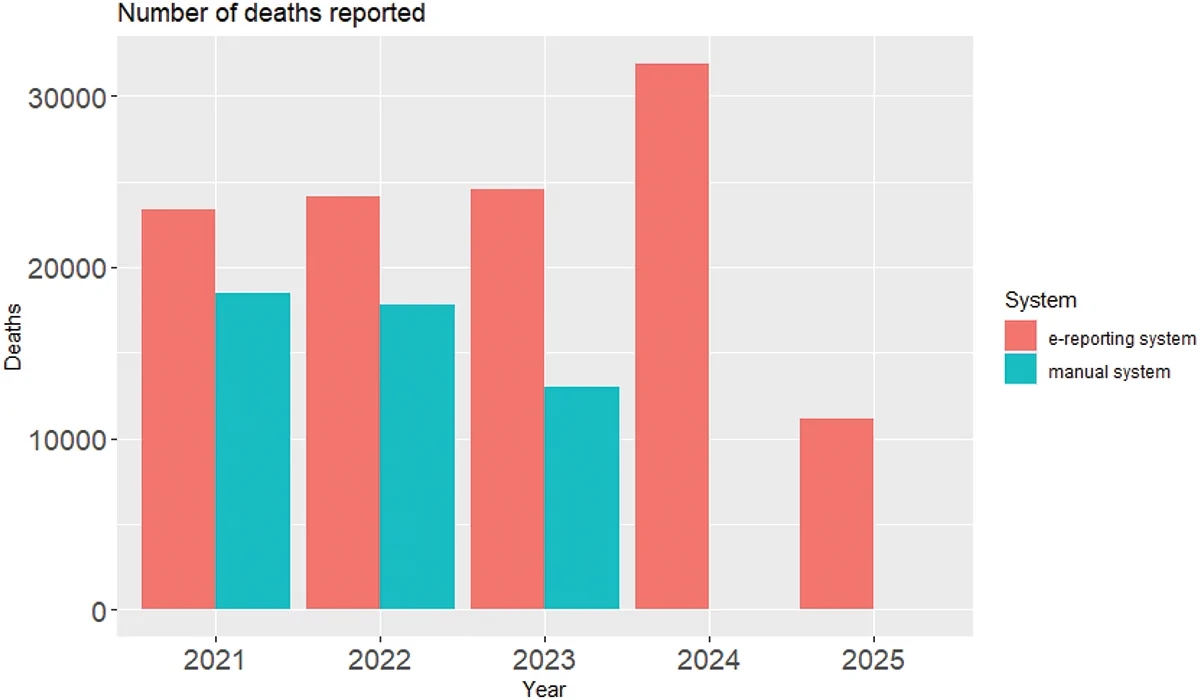
Yearly distribution of number of deaths captured through the manual system in comparison to the e-reporting system for the period of January 2021 to June 2025.

#### Data quality monitoring

The real-time death reporting dashboard ensured continuous monitoring of the data flow and this flag any inconsistencies. Components of this dashboard are shown in Figure 1.

#### Data quality of age values between both systems

When assessing age-related data quality, we found that the manual system contained substantial outliers, including ages over 250 years, revealing significant data entry errors. The e-reporting system, however, demonstrated a marked improvement in data quality, largely because it was designed with enhanced quality checks integrated directly into the data entry process. Additionally, the e-reporting system proved more comprehensive, capturing a wider range of data (IQR = 45) with a lower probability of missing information in specific age groups compared to the manual system through INRIS (IQR = 42) (Figure 4). Ultimately, the improved quality and constant availability of real-time data from the e-reporting system facilitates monitoring and quality assurance.

**Figure 4.**
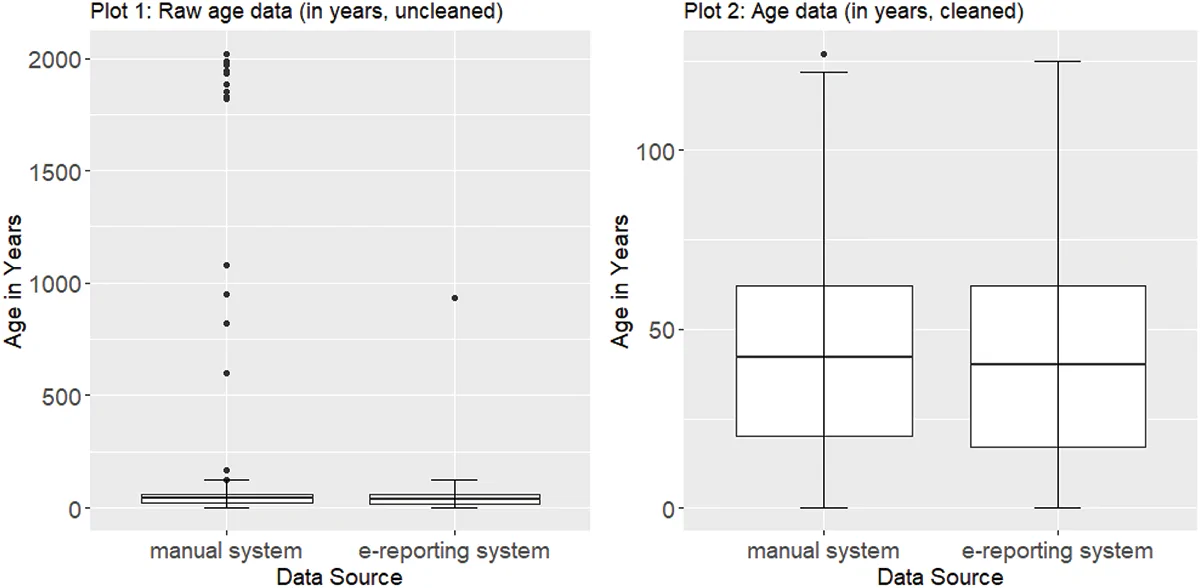
Data quality in age (original data) and data quality in age (cleaned data); plot 1 showing boxplots of age (in years) raw values with presence of outlier points in comparison with plot 2 having cleaned age value.

#### Completeness and timeliness

The introduction of the e-reporting system significantly addressed long-standing issues of completeness and timeliness.

##### Completeness

The e-reporting system captured a higher proportion of deaths, achieving an estimated rate of 17.4% (95% CI: 17.3%−17.5%). This marks a notable improvement over the manual system, which recorded a lower completeness rate of 11.9% (95% CI: 11.8%−12.0%). Statistical analysis confirmed a significantly higher number of deaths recorded by the electronic system compared with the manual system (chi2 = 4.01, *p* < 0.045).

##### Timeliness

The e-reporting system also proved significantly more efficient. The average reporting time from the date of death was approximately 3 days, with a variance of 24 days. This represents a statistically significant reduction compared to the manual system, which had an average reporting time of 6 days and a variance of 62 days (*p* < 0.001) (Figure 5).

**Figure 5.**
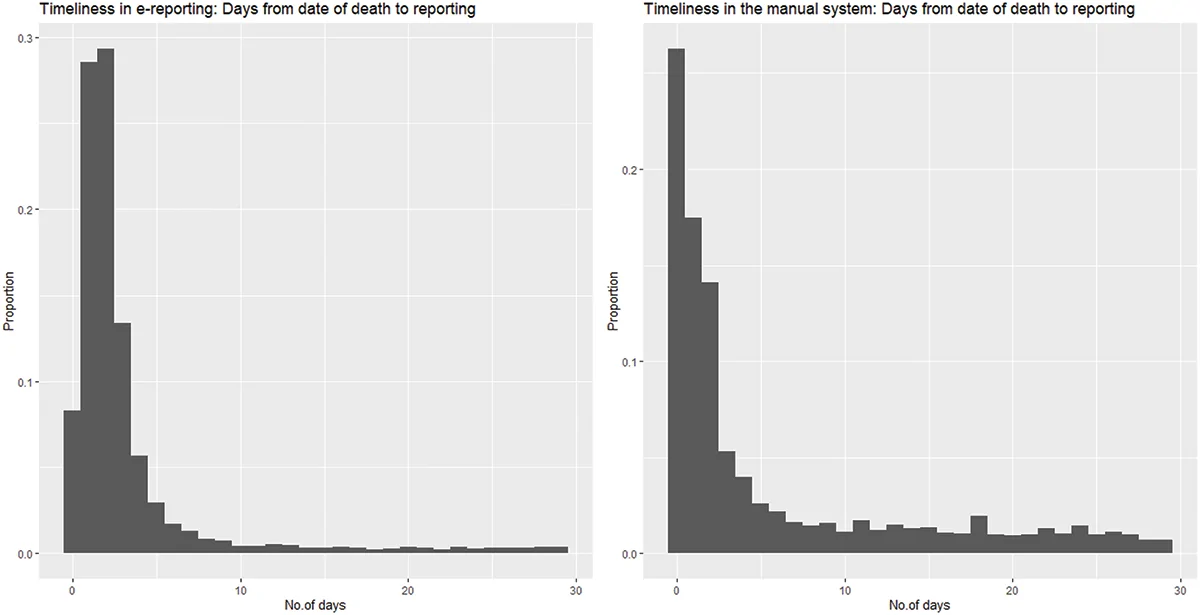
Reporting timeliness comparison between e-reporting system and the manual system.

## Discussion

### Interpretation of digital implementation success

The digital implementation case study suggests that integrating electronic data collection and analysis platforms into CRVS systems can improve the completeness and timeliness of death notification in LMICs like Zambia. The observed increase in reported deaths (17.4% completeness) using this system is consistent with successful CRVS digitization initiatives in other developing countries, such as Papua New Guinea and Tanzania [8]. Despite the improvements associated with electronic reporting, overall completeness remained below 20%, indicating that while digital reporting can improve the efficiency and quality of the notification process, achieving high completeness will require broader strengthening of the death notification process through interventions such as proactive community-based notification through local authorities and community health workers, stronger linkage and reconciliation of health facility, burial and civil registration records, and integration of e-reporting with INRIS. While the absolute completeness rate remains modest relative to international targets, the 50% reduction in reporting time (6 days to 3 days) is a critical operational achievement. This acceleration creates the potential for public health officials to access and act upon mortality data more rapidly.

The success is attributable to the core features of the digital platform: real-time data capture using mobile technology, standardized data collection via the digitized Form 13, and the integration of automated quality checks. The real-time dashboard further supports the WHO’s recommendation that digital tools be a key component of modern CRVS strengthening strategies [14].

### Scalability, cost implications, and sustainability

The utilization of an accessible, open-source tool like KoboToolbox hosted on MoH servers demonstrates the potential for scalable implementation across all remaining districts. Beyond the immediate study sites, the digitized death reporting system provides a scalable model for strengthening other components of Zambia’s CRVS and health information systems. This includes the Ministry of Local Government-Local Council offices, who are among the key stakeholders in death registration process in Zambia in charge of burial permits and burial sites. Given that many countries face similar challenges of delayed and incomplete death reporting, the operational approach described here may be relevant to other jurisdictions seeking incremental, sustainable pathways to modernizing CRVS systems.

Our findings support a phased transition toward electronic death notification rather than immediate replacement of all paper-based processes. The components most suitable for early digitisation are the capture, validation, submission, archiving, and transmission of Form 13, particularly where these processes currently depend on manual data entry and physical transportation of forms. Further integration of the electronic notification platform with INRIS would help reduce duplication and facilitate continuity between death notification and civil registration. Paper documentation may, however, remain necessary for components of the burial and legal registration processes until the required regulatory, infrastructure, and interoperability arrangements for fully electronic processes are established.

The transition from paper-based, transport-intensive reporting to a digital submission model that requires only mobile data, has the potential to reduce some operational burdens associated with the transportation, processing, and manual entry of notification form over time. Furthermore, hosting the platform within the Ministry of Health infrastructure strengthens government ownership and long-term sustainability by reducing dependence on externally hosted, vendor-managed systems.

### Limitations

While this study offers evidence of operational success, it has several limitations. First, data collection was centralized in selected high-mortality provinces (59.8% in Lusaka), potentially introducing coverage bias that may not fully represent the entire country’s reporting challenges. Second, the e-reporting system is not yet fully integrated into the central INRIS. Full integration is essential to realize maximum efficiencies and reduce potential duplication between electronic notification and civil registration processes. However, our findings provide the necessary evidence to support further integration and strengthening of the electronic notification component of the CRVS system. Third, the study used historical manual reporting data as a reference for comparison with electronic reporting period thus multivariate analysis to control for temporal differences and other potential confounding factors such as changes in staff workload and seasonal reporting patterns was not performed and is beyond the scope of this case study. Fourth, analysis of cause of death (COD) data was not included in this manuscript, as the primary objective was to evaluate the impact of implementing a mobile-based platform for digitizing death reporting. Furthermore, community deaths, unlike those occurring in health facilities, typically lack medically certified causes of death, limiting the feasibility of reliable COD analysis within this context. Finally, the reliance on passive reporting from families visiting burial offices means some community deaths may still be missed, highlighting the need for a more proactive, community-level death surveillance model.

### Future directions

Future research should focus on three key areas:

1. Conducting a formal cost and cost-effectiveness analysis of the digital system compared with the full operating costs of the manual system, including infrastructure, data transmission and system maintenance.
2. Conducting an implementation study to assess the challenges and benefits of full integration of the e-reporting system into the INRIS platform.
3. Developing and evaluating a proactive, community-based death reporting model that leverages the local authority offices and community health workers to address the coverage gaps with the current passive reporting model and support sustainable scaling to other provinces not covered during the pilot study.

## Conclusions

The implementation of the electronic death notification system in Zambia represents a significant step towards strengthening the national CRVS system. This digital intervention successfully achieved an estimated completeness rate of 17.4% (95% CI: 17.3%−17.5%) and reduced the average time to report a death from 6 days to 3 days (*p* < 0.001). The prompt capture of high-quality death reports reduces the chances of missing deaths and enhances the ability to track disease trends, directly supporting Sustainable Development Goal 3 (Good Health and Well-being). Although this study focused specifically on mortality reporting, improved completeness and timeliness of death registration, this effort can also strengthen the broader civil registration and identity management system, contributing indirectly to SDG 16.9 on legal identity.

We explicitly recommend the immediate and full integration of this implemented e-reporting platform into the Department of National Registration, Passport and Citizenship’s INRIS to ensure nationwide scale-up to improve overall reporting and registration coverage, maximize operational efficiency, and provide reliable, real-time data for public health policy and planning [16]. In addition, we also encourage inter-government agency collaboration to improve death reporting and notification coverage.

## Supplementary Material

Digitizing death reporting in Zambia_Supplementary_22082026.docx

## Data Availability

The datasets generated and/or analysed during the current study are not publicly available due to the sensitivity of the data; however, they may be made available upon reasonable request from the Ministry of Home Affairs through official channels of the Government of Zambia and the Department of National Registration, Passports and Citizenship (DNRPC).
