# Supplementary material for: Digitizing death reporting in Zambia: evaluation of a mobile-based civil registration system
Source: Glob Health Action. 2026 Sep 28;19(1):2732479. doi: 10.1080/16549716.2026.2732479 (PMC13629807; doi:10.1080/16549716.2026.2732479)
Supplement: Supplementary file 1 [file ZGHA_A_2732479_SM3246.docx]

**Digitizing death reporting in Zambia: Evaluation of a mobile-based civil registration system**

**Supplementary Tables**

**Supplementary Table 1**: Distribution of notified deaths across all age groups and provinces captured through the e-reporting system in Zambia, July 2024 - June 2025

| Characteristics | *N* = 115573 | |
| --- | --- | --- |
|  | **n** | **%** |
| Age Groups | | |
| 0 yrs | 18,884 | 16.3 |
| 01-4 yrs | 5,158 | 4.5 |
| 05-<9 yrs | 1,977 | 1.7 |
| 10-<14 yrs | 1,758 | 1.5 |
| 15-<19 yrs | 2,575 | 2.2 |
| 20-<24 yrs | 4,241 | 3.7 |
| 25-<29 yrs | 5,994 | 5.2 |
| 30-<34 yrs | 7,622 | 6.6 |
| 35-<39 yrs | 7,858 | 6.8 |
| 40-<44 yrs | 8,432 | 7.3 |
| 45-<49 yrs | 7,425 | 6.4 |
| 50-<54 yrs | 6,538 | 5.7 |
| 55-<59 yrs | 5,290 | 4.6 |
| 60-<64 yrs | 5,640 | 4.9 |
| 65-<69 yrs | 5,448 | 4.7 |
| 70-<74 yrs | 5,405 | 4.7 |
| 75-<79 yrs | 5,174 | 4.5 |
| 80-<84 yrs | 4,458 | 3.9 |
| 85+ yrs | 5,696 | 4.9 |
| Province | | |
| Central | 7,518 | 6.5 |
| Copperbelt | 31,652 | 27.4 |
| Eastern | 8 | <0.1 |
| Luapula | 7 | <0.1 |
| Lusaka | 69,076 | 59.8 |
| Muchinga | 7 | <0.1 |
| North | 1,779 | 1.5 |
| Northern | 4 | <0.1 |
| Southern | 3,148 | 2.7 |
| Western | 2374 | 2.1 |

**Supplementary Figures**

**Supplementary Figure 1**: Schematic flow of manual death notification and registration flow in Zambia.
